# Paper-Based Microfluidic Chips for Food Hazard Factor Detection: Fabrication, Modification, and Application

**DOI:** 10.3390/foods12224107

**Published:** 2023-11-13

**Authors:** Meiqi Liang, Guozhi Zhang, Jie Song, Mingqian Tan, Wentao Su

**Affiliations:** 1 Academy of Food Interdisciplinary Science, School of Food Science and Technology, Dalian Polytechnic University, Dalian 116034, China; 13840657193@163.com (M.L.); l15140757730@163.com (G.Z.); jiesongdpu@163.com (J.S.); mqtan@dlpu.edu.cn (M.T.); 2 National Engineering Research Center of Seafood, Dalian 116034, China; 3 SKL of Marine Food Processing & Safety Control, Dalian Polytechnic University, Dalian 116034, China

**Keywords:** food safety, microfluidics, paper-based device, detection method

## Abstract

Food safety and quality are paramount concerns for ensuring the preservation of human life and well-being. As the field of food processing continues to advance, there is a growing interest in the development of fast, instant, cost-effective, and convenient methods for detecting food safety issues. In this context, the utilization of paper-based microfluidic chips has emerged as a promising platform for enabling rapid detection, owing to their compact size, high throughput capabilities, affordability, and low resource consumption, among other advantages. To shed light on this topic, this review article focuses on the functionalization of paper-based microfluidic surfaces and provides an overview of the latest research and applications to colorimetric analysis, fluorescence analysis, surface-enhanced Raman spectroscopy, as well as their integration with paper-based microfluidic platforms for achieving swift and reliable food safety detection. Lastly, the article deliberates on the challenges these analytical methods and presents insights into their future development prospects in facilitating rapid food safety assessment.

## 1. Introduction

Food safety and quality are paramount concerns for ensuring human lives and well-being. Addressing the complexities of this issue necessitates a comprehensive understanding of the various factors that can compromise food safety. These factors, classified based on their origin and characteristics, include foodborne pathogenic bacteria, environmental contaminants (e.g., heavy metal ions, persistent organic pollutants), unauthorized or abusive use of food additives, contamination during food production (such as pesticide residues and veterinary drugs), toxins present in food raw materials (e.g., mold, plants, marine algae), and food allergens [1]. Developing efficient and sensitive technologies for detecting potential harmful elements at each stage of the food industry chain holds significant practical implications for safeguarding public health and preventing life-threatening situations [2]. 

Currently, five conventional methods are available for testing hazardous substances in food: gas chromatography [3], high-performance liquid chromatography [4], mass spectrometry [5], polymerase chain reaction [6,7], and immunoassays [8,9,10]. However, these detection methods mostly rely on instrumental analysis, which although accurate and reliable, have limitations such as expensive instruments, long cycles, large physical consumption, and the requirement of professionals to operate, making them unsuitable for real-time, fast, and portable needs of on-site food detection. In light of this, there is a pressing need for the development of food safety testing technologies that can meet the on-site, fast, accurate, and public types. The development of such testing technologies will be crucial in ensuring food safety and protecting public health. 

In recent years, microfluidic chip technology has emerged as a promising field, exhibiting unique advantages such as precise control of microscale liquid flow, low reagent consumption, rapid analysis speed, multi-functional integration, and high throughput. This technology has found application prospects across various domains of life sciences, including rapid bioanalysis, biochemical testing, and pathogen detection [8,9,10,11]. Notably, paper-based microfluidic technology has demonstrated remarkable development potential and promising applications in the arena of rapid food safety detection. It offers numerous advantages, including cost-effectiveness, facile chemical modification, and independence on external power sources. Furthermore, the combination of microfluidic paper chip analysis sensing methods with diverse instruments/equipment and personal electronic devices (e.g., smartphones) has emerged as a notable research trend [8]. This article primarily focuses on the preparation strategy, analysis methods, and the utilization of integrated microfluidic paper chips in the context of rapid food safety detection. The authors also envision the future development prospects and challenges for this technology in the domain of fast analysis and detection of food safety issues.

## 2. Fabrication Strategy of Paper-Based Microfluidic Chips

Paper-based microfluidic chips, a burgeoning technology, involve the infiltration of hydrophobic materials into hydrophilic paper fibers using various techniques. This enables the controlled flow of fluids within the paper fibers through the hydrophobic “walls”, resulting in the creation of paper-based microfluidic chips. The selection of suitable paper is critical for chip production. The chosen paper must possess sufficient mechanical strength to resist deformation in liquid environments, exhibit appropriate hydrophilicity or hydrophobicity to form test regions and demonstrate chemical stability without reacting with samples. Currently, filter paper serves as the preferred substrate material due to its excellent water absorption, resistance to deformation, affordability, and availability [12,13,14,15]. Additionally, filter paper features a smooth, flat surface with uniform properties, ideal fluid flow rates, and high particle retention. Common options include Whitman series filter papers and chromatographic papers. Different paper types can be selected based on specific testing requirements.

The pivotal step in paper chip fabrication involves constructing a hydrophilic network to control liquid flow, resulting in two-dimensional (2D) paper chips. Three-dimensional (3D) paper chips can also be achieved through techniques such as stacked bonding or origami [16,17]. Each method possesses distinct advantages and disadvantages, yielding paper chips with varying characteristics. Consequently, different processing methods can be adopted based on specific chip requirements. The construction of hydrophilic channels can be categorized as follows: the formation of hydrophobic channels on paper using materials like paraffin, photoresist, or urethane acrylates (not applicable for organic solvents); blocking of paper holes with polymers such as poly-dimethicone (PDMS), polystyrene, or polyurethane to create physical barriers, subsequently establishing channels through physical cutting [18].

### 2.1. Wax Printing Method

Currently, the most widely used method for producing microfluidic chips is wax printing. This method is preferred due to the low cost and easy availability of wax, as well as its low chemical reactivity. Wax printing involves heating the wax and allowing it to penetrate a piece of paper, which is then printed using a wax spray printer [19]. One advantage of this method is the ability to produce paper chips in large quantities, although the resolution of the patterns is not high. Different thicknesses and quantities of wax can be used to create closed, semi-closed, and varying-sized channels on the paper. Alternatively, hand drawing with a crayon can be used to create paper chips with simple patterns and a low resolution, without the need for organic solvents. Screen printing is a simple method for producing large-scale paper chips [20]. The fusion penetration method does not require instruments or chemical reagents, but the accuracy of the resulting paper chip is low [21]. The flexo printing method can quickly produce a large number of paper chips, but it requires a specialized flexographic printing machine and a single-plate printer [22]. 

**Figure 1:**
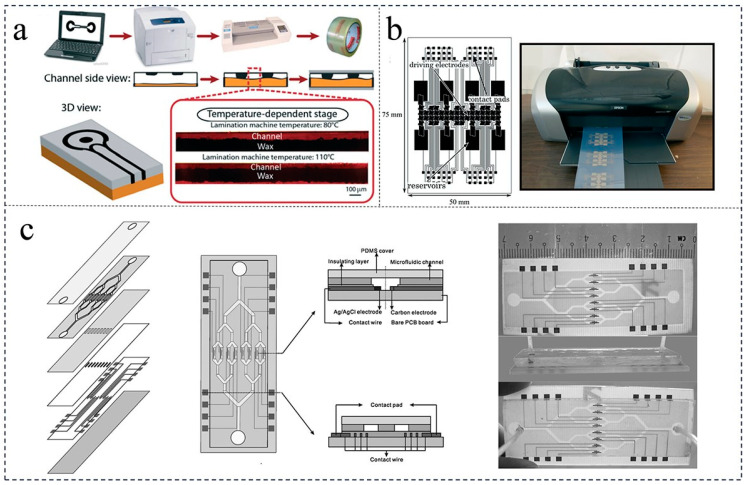
Printing methods used for microfluidic chip fabrication. (**a**) Wax printing method [27] (reproduced with permission from publisher Royal Society of Chemistry). (**b**) Inkjet printing method [27] (reproduced with permission from publisher Royal Society of Chemistry). (**c**) Screen printing method [33] (reproduced with permission from publisher Royal Society of Chemistry)**.**

Three-dimensional (3D) printing methods are commonly used for processing microfluidic chips or the inverted back molds of microfluidic chips. These methods include laser printing [23], inkjet printers [24,25], wax printers [26,27], and screen printing [28] (Figure 1). Three-dimensional-printed microfluidic chips are commonly used in paper-based microfluidic chips due to the hydrophobicity inks that form microchannels in the hydrophilic paper material [29]. The pattern accuracy is determined by the precision of the printer or screen mesh, and typically ranges from 80 to 400 μm. During the fabrication process of the chip, the patterned area consisting of hydrophobic inks is cured to produce the desired 3D shape. The experimental and numerical analysis of three Y-shaped split and recombination micromixers is based on cantor fractal structures [30]. If a conductive ink containing silver nanoparticles is used, electrodes can be printed on the surface of the microfluidic chip [31,32]. The basic processing model for screen printing is illustrated in Figure 1, where microchannels and silver electrodes are processed using a screen printing method based on a UV-sensitive media slurry [33]. Additionally, a novel printing method is employed for the electrodes.

The main methods for processing microfluidic chips using 3D printing are stereolithography and fused deposition modeling (FDM) [34,35]. Fused deposition modeling 3D printers are commonly used for producing low-cost D micro3D microfluidic chips. This method allows for the direct printing of materials such as PC, PLA (polylactic acid), ABS (acrylonitrile), and others for manufacturing three-dimensional microfluidic chips [36]. It can also be used to print PDMS mold molds. However, commercial fused deposition modeling has an accuracy range of 100–500 μm and a limited selection of transparent printing consumables suitable for microfluidic chips. The processing speed of chips using this method is slower compared to other methods.

### 2.2. Etching Method

**Figure 2:**
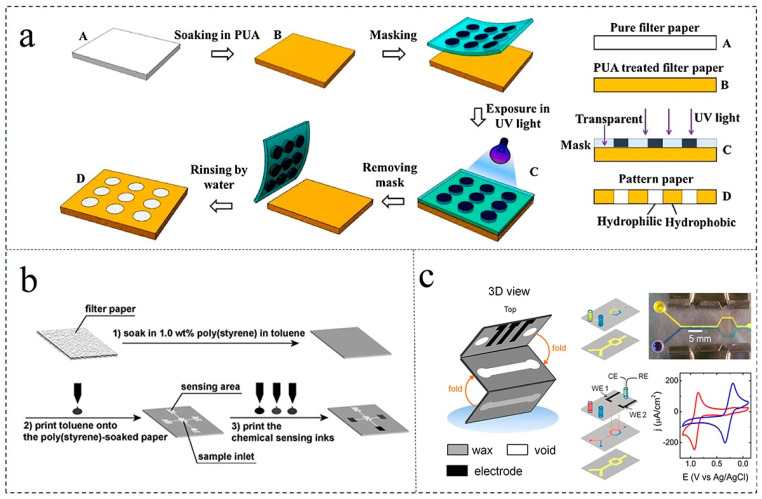
Steps to make a paper chip. (**a**) Photolithography [39] (reproduced with permission from publication Sensors and Actuators B: Chemical); (**b**) etching method [40] (reproduced with permission from publisher American Chemical Society); (**c**) cutting method [45] (reproduced with permission from publisher American Chemical Society).

Etching, as a chemical modification technique, is employed to transform the hydrophobic layer of paper into a hydrophilic one [37]. In contrast, lithography, a production method, utilizes materials like photoresist or negative photo adhesive SU-8 to establish a hydrophobic barrier, enabling the creation of high-resolution patterns. For instance, Li et al. successfully achieved well-defined and stable patterned paper chips by utilizing ultraviolet light to degrade self-assembled silanized monolayers [38]. SU-8 photoresist, an epoxy-type near-ultraviolet negative photoresist, can be directly deposited on a paper substrate to form hydrophilic microstructural channels through photolithography [39], inkjet printing, or screen printing [40,41] (Figure 2). However, the production of negative light adhesive SU-8 is intricate and expensive, and its low flexibility contributes to the fragility of the paper chip [42]. To enhance flexibility while maintaining low cost and environmental friendliness, Han et al. substituted SU-8 with a photosensitive material called polyurethane acrylate (PUA) [43].

Another method for restoring local hydrophilicity in paper is the plasma method, which involves the use of plasma-emitting equipment. Recently, Kao et al. developed a battery-powered portable plasma generation device that can chemically modify the hydrophobic region of paper into a hydrophilic one under normal conditions [44]. This portable plasma device eliminates the dependence on large plasma equipment, thus making the production of paper chips more convenient and efficient.

### 2.3. Cutting Method

The cutting method is a technique used to facilitate the formation of paper chips by creating physical boundaries in the form of channels or patterns. Two primary cutting methods are employed: process cutting and laser cutting. Process cutting involves computer-operated cutting, which takes into account various cutting strengths and angles to generate paper chips. Additionally, a protective layer can be added at the bottom to prevent damage to the paper pieces. On the other hand, laser cutting does not necessitate an additional protective layer but requires specialized laser cutting instruments, making it a more challenging technique to execute. Crooks et al. have demonstrated that laser cutting can significantly enhance fluid velocity by creating designed channels, resulting in sandwich paper chips with hollow middles [45]. Specifically, laser ablation refers to a method of ablating and processing microfluidic channels on the surface of polymeric materials using a carbon dioxide laser with a wavelength of 10 μm [46]. This technique offers the advantages of simplicity, speed, and one-time ablation for processing microfluidic channels. It finds widespread application, as most polymeric materials and glasses can utilize this method to process microfluidic channels on their surfaces. However, a drawback of laser ablation is the uneven processing of the inner wall of the microchannel on the polymer material’s surface, leading to the presence of numerous bubbles that may further require chemical treatment [47]. Furthermore, bulges formed by the casting and resolidification of molten material are present on both sides of the flow channel processed on the polymer material’s surface, hindering effective bonding. Moreover, the processing accuracy is limited, making it suitable only for objects with flow channels wider and deeper than 80 μm. While lasers are primarily applied to single-polymer materials for the development of low-cost microfluidic chips, there is ample room for future advancements in the processing of biodegradable plastics, paper, conductive plastics, and other materials in the field of microfluidic chip fabrication.

Because of its simple operation, low cost, and large number of preparations, the wax printing method is widely used in the fabrication of hydrophilic and hydrophobic channels on paper-based chips, and the method can be completed by hand-drawing or paraffin printers. However, the on-chip pattern obtained by the wax printing method has low resolution. Similarly, screen printing can also produce paper in large quantities, but the resulting chips are less accurate and require specialized printing presses to complete the production. Although paper-based chip patterns prepared using etching have high resolution, the photoresist materials used are complex, expensive, and inflexible to produce. The process cutting method in cutting method uses a computer to operate, which can cut the paper according to different cutting strengths and angles, but add a protective layer in order not to destroy the paper. Although laser cutting can solve this problem, the processing accuracy of laser cutting is limited, and it is only suitable for objects with flow channel widths and depths greater than 80 μm, and laser cutting is currently mainly used for single polymer materials.

## 3. Surface Functionalization Modification of Paper-Based Chips

Paper-based surface functionalization modification serves as the foundation for the development of paper-based platform analysis methods. The selection of paper type plays a crucial role in the production of paper chips, with Whatman No.1 filter paper being the most commonly utilized type due to its composition of 98% α-cellulose, providing a smooth and flat surface, uniform properties, moderate fluid flow rate, and high particle retention. Other types of paper, such as nitrocellulose paper, wax paper, glass fiber paper, and cotton paper, fiber paper, are also frequently employed [48].

### 3.1. Electrostatic Adsorption Method

One method used for surface functionalization and modification is the electrostatic adsorption method, which relies on electrostatic attraction and van der Waals forces to adsorb materials onto the paper [49]. To achieve the desired surface coating, the solution containing the sensing material can be soaked or directly applied to the paper and allowed to dry [50,51]. Cellulose paper, commonly used in this context, contains polysaccharide structures that contribute to its composition, including hydroxyl groups or negative charges [52]. As a result, it typically only adsorbs cations and positively charged molecules. However, for the modification of fixed biomolecules, a higher surface density of negative charges is required. To address this issue, cross-linking or surface coatings agents are necessary to enhance the adsorption capacity of paper-based substrates [53,54]. In recent years, researchers have made advancements in surface functionalization and modifications of paper chips to improve the fixation of functional molecules and particles onto the paper matrix.

### 3.2. Covalent Bond Modification Method

The presence of functional groups, such as hydroxyl groups and reducing ends of cellulose rings, on cellulose paper allows for chemical modification of the paper surface, thereby enhancing the abundance of active groups in the paper chip. Among these functional groups, the hydroxyl group is most likely to undergo reactions. The activity of the hydroxyl group varies depending on its involvement in intramolecular hydrogen bonding, with the lowest activity observed at position C3, while the highest activity is observed at positions C2 and C6 [55]. These functional groups can effectively stabilize the sensing material on the paper surface through the formation of covalent bonds. Several chemical modification methods have been developed to ensure the stable attachment of sensing materials to paper-based surfaces. For instance, oxidation of the paper can generate carbonyl or carboxyl groups, which can then be covalently bonded to molecules containing amino groups. Moreover, the etherification of cellulose hydroxyl groups and the formation of epoxy groups through the reaction of glycidyl methacrylate can immobilize the sensing material [56]. Carboxyl esterification of hydroxyl groups can also modify cellulose paper, enabling the fixation of azo dyes. Cationic nitrocellulose paper can be hydroxy-esterified with HNO_3_ in the presence of strong acids like H_3_PO_4_ or H_2_SO_4_. Additionally, the modification of paper can be achieved by incorporating cationic polymers, such as polyamide–epichlorohydrin and polyvinyl alcohol, to enhance molecular bonding efficiency.

### 3.3. Embedding Method

The embedding technique involves the incorporation of biomolecules into a specific matrix using the sol-gel method, resulting in the production of bioactive paper. This method primarily entails three steps: firstly, the sol-gel containing the biomolecular material is applied to the paper through inkjet printing; secondly, a silica-containing substrate layer is formed within the system; and finally, the material is firmly attached to the paper with the assistance of this silica layer [57]. The silicon layer serves as an intermediary during this process. Moreover, the sol-gel method can also be employed to create a modification layer [58]. Wang et al. demonstrated the embedding of an enzyme between the polymer (polyarginine) and silica layers, and the use of multilayer embedding ensures the stability of paper fiber modification and enzyme activity [59].

Electrostatic adsorption requires cellulose paper to contain a polysaccharide structure that can adsorb cationic and positively charged molecular substances through electrostatic attraction and van der Waals forces. However, due to the insufficient negative charge density on the surface of cellulose paper, additional crosslinkers or surface coating agents need to be added to enhance the adsorption capacity of the paper-based substrate. The covalent bond modification method can rely on the chemical reaction between the functional groups in the cellulose paper and the sensing material to form covalent bonds, to realize the modification of the paper. Unlike the first two modification methods, the embedding technique requires not only the incorporation of sol-gel into the paper, but also the assistance of a silica layer.

## 4. Analysis Method of Paper-Based Microfluidic Chips

The analysis methods employed for the detection of food safety in microfluidic paper chips primarily consist of electrochemical analysis, fluorescence analysis, colorimetric analysis, and the emerging surface-enhanced Raman method (SERS).

### 4.1. Electrochemical Method

The electrochemical method utilizes a three-electrode system consisting of a working electrode, a reference electrode, and a counter electrode. By establishing a current path in the electrolyte, the working electrode and the counter electrode enable sample detection based on electrochemical signals. This method possesses several notable characteristics, including low cost, easy operation, high selectivity, high sensitivity, and excellent portability. Moreover, it demonstrates exceptional compatibility with paper chips. Currently, electrochemical paper chips have extensive applications in the domain of food safety testing. For instance, Wang et al. developed a disposable paper-based electrochemical sensing platform that utilizes the unique physical and chemical properties of graphene nanosheets and gold nanoparticles to achieve sensitive detection of nitrite [60]. Similarly, Guadarrama-Fernández et al. developed an innovative paper-based bioelectrochemical sensor for the detection of glucose content in beverages. This sensor employs platinum paper as the working electrode carrier and a biocompatible polymer membrane containing a mixture of polyvinyl alcohol and chitosan, which incorporates glucosidase oxidase as the recognition layer. By utilizing potential detection, this sensor enables rapid detection of glucose content in orange juice, with a detection sensitivity of (119.6 ± 6.4) mV/dec, a detection range of 0.03~1.0 mmol/L, and a detection limit of 0.02 mmol/L. Furthermore, electrochemical paper chips can be assembled into 3D structures through origami techniques, thereby providing an enhanced analysis platform for rapid food safety detection [61].

### 4.2. Optical Analysis

**Figure 3:**
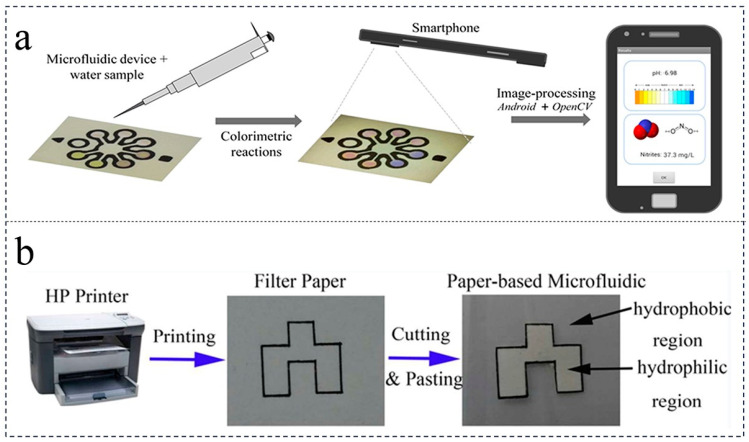
Example of a paper chip that detects heavy metal ions by colorimetric analysis. (**a**) Paper-based microfluidic devices detect nitrite concentration and pH, and display the results in an Android application [62] (reproduced with permission from publisher American Chemical Society). (**b**) The manufacturing process of paper-based microfluidic devices [63] (reproduced with permission from publisher RSC Pub).

The colorimetric analysis process employed in paper-based platforms typically involves the application of a small droplet of the sample solution onto the platform. Utilizing capillary force and gravity, the sample solution is directed in an organized manner towards the hydrophilic channel, where it interacts with chromogenic reagents, resulting in observable color changes. These color formations or alterations are then utilized for qualitative and quantitative analysis of the target substance. The color signal can be conveniently observed with the naked eye or captured through photography or scanning [62,63,64] (Figure 3). The detection of nitrate and nitrite substances in food is of utmost importance due to their potential to generate harmful compounds when reacting with amines present in food. Consequently, researchers have developed paper chips based on colorimetric analysis to detect nitrate and nitrite. Trofimchuk et al. successfully created a microfluidic paper chip colorimetric analysis device, which employed the Griess reagent to determine nitrite concentration based on the resulting color change. Furthermore, the utilization of the coffee ring effect enhanced the sensitivity of the detection process. This device achieved the detection of meat nitrite content within a short duration of 15 min, with a detection limit of 1.1 mg/kg [60]. In addition to nitrates and nitrites, the excessive presence of heavy metal ions such as lead in food poses significant health risks. Previous studies have used paper-based chips combined with colorimetric methods to detect heavy metal ions [65]. Our research group has demonstrated the detection of heavy metal ions, including lead, in food using colorimetric analysis on paper chips, which exhibited notable characteristics such as high sensitivity and efficient testing outcomes. Moreover, our group has implemented parallel detection technology to simultaneously analyze various heavy metal, ions, resulting in time-saving, cost-effective, and significantly improved analysis efficiency. The advantages of colorimetric methods over electrochemical techniques lie in their ability to conduct preliminary analysis with the naked eye, without the need for specialized instruments. This analysis method is relatively straightforward and user-friendly.

**Figure 4:**
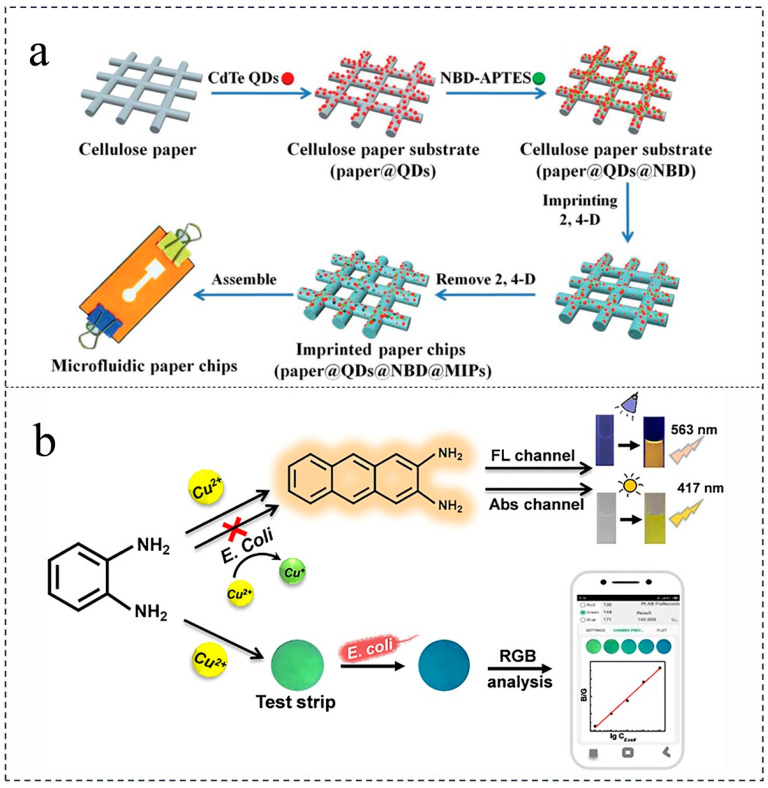
(**a**) The preparation process of paper-based microfluidic proportional chips [66] (reproduced with permission from publisher Royal Society of Chemistry). (**b**) Schematic diagram of the detection of *E. coli* via fluorescence with phone analysis [68] (reproduced with permission from publication Analytical and Bioanalytical Chemistry).

Fluorescence techniques necessitate the utilization of a fluorometer for analysis. Typically, fluorescence quenching of bound fluorophores on the surface of the specimen to be examined or quantum dots on paper is employed for detection. In the context of expedited food safety assessment, the interaction between the target and the fluorescent probe is crucial. By monitoring the alteration in fluorescence signal emitted by the fluorescent probe, the presence of the target substance can be determined. In their study, Zhang et al. devised an innovative molecularly imprinted fluorescence sensing microfluidic paper chip utilizing an electron transfer-induced fluorescence quenching mechanism, enabling the specific identification and sensitive detection of the pesticide 2,4-dichlorophenoxyacetic acid [66] (Figure 4). Beyond pesticide identification and detection, fluorescence analysis also plays a significant role in food testing. Adkins et al. have developed a detection platform that combines transparencies-based electrochemical analysis and paper-based colorimetric analysis for the rapid analytical detection of *E. coli* and enterococcal metabolites in food and water [67]. Wang et al. established an intelligent fluorescence and colorimetric dual-reading sensing system to achieve simple and rapid detection of *E. coli* [68]. The fluorescence method offers several advantages, including high selectivity and sensitivity, low detection limit, good repeatability, and minimal sample requirements. However, it necessitates instrumental assistance and may be susceptible to interference from fluorescent agents present in the paper substrates.

SERS is a molecular vibrational spectroscopy technique that amplifies the Raman spectral signal of molecules, leading to improved detection levels and lower detection limits. In SERS sensing analysis, nanoparticles are commonly employed as the SERS substrate, resulting in a significant enhancement (10^4^~10^12^ times) of the Raman scattering signal of the target analyte. Consequently, SERS enables the detection of biochemical substances at extremely low concentration levels. Notably, the presence of a three-fiber-dimensional fiber matrix in paper materials enhances the SERS effect. This property has made SERS widely applicable in the detection of pesticide residues, such as methyl parathion, which often exhibit distinct Raman characteristic peaks. Xie et al. successfully synthesized gold nanoparticles through seed-mediated growth and subsequently immobilized them on filter paper via an impregnation method [69]. By utilizing a portable Raman spectrometer, they achieved a detection limit of 0.011 μg/cm^2^ for methyl parathion. Furthermore, this method was employed for the rapid detection of melamine in diluted milk, with a detection time of only 10 s, a detection limit of 1 mg/kg (ppm), and a linear range of 1~1000 mg/kg (ppm). The SERS paper-based platform offers several advantages, including its simplicity in preparation, reusability, scalability, and compatibility with droplet detection. However, certain limitations exist due to the anisotropic and heterogeneous structure of paper fibers, which results in uneven adsorption of nanoparticles and the formation and distribution of plasma hot spots in a random manner [70]. Raman signal is primarily collected within a laser diameter area of approximately 5 μm.

## 5. Application of Integrated Paper-Based Microfluidic Chips for Rapid Food Safety Detection

In the contemporary era, the fundamental requirement for human beings to maintain good health is the consumption of safe food. The focus on food quality and safety has always been of utmost importance. The factors influencing food safety can be categorized based on the source and nature of the food, including self-food ingredients, pesticide residues, pathogenic bacteria, heavy metal content, and food additives, among others. The analysis of large quantities of food, particularly for food analysis, presents challenges in using conventional large instruments and equipment for routine testing. Microfluidic paper-based analysis has gained attention significant attention due to its rapid, efficient, user-friendly, and cost-effective characteristics (Table 1).

**Figure 5:**
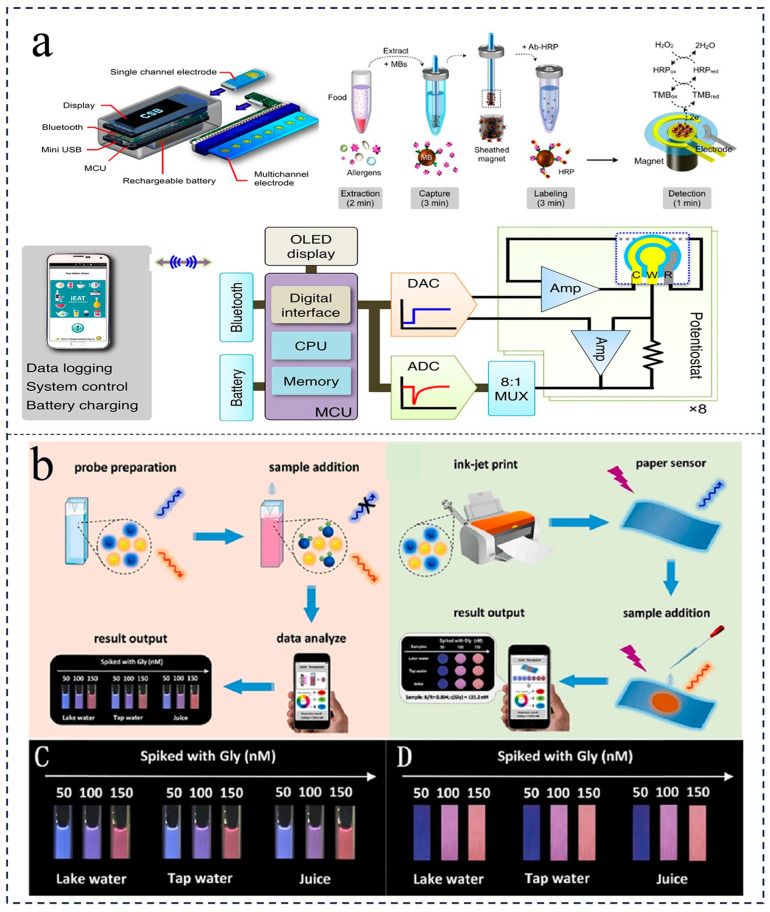
(**a**) Integrated microfluidic magneto-chemical systems for allergens in foods [73] (reproduced with permission from publisher American Chemical Society). (**b**) Integrated microfluidic photochemical systems for the detection of hazardous substances in foods [89] (reproduced with permission from publication Journal of Hazardous Materials).

The integration strategy of the active mode primarily encompasses two systems: centrifugal and micro-pump microvalves. In 2012, Chen et al. introduced the centrifugal disc laboratory, which enables the automated detection of allergens within a 30-min timeframe [71]. Centrifugation serves as a straightforward method for liquid integration, achieved through the spinning of a disk sample for sample storage and transport. The design of the equipment, combined with the appropriate rotation speed, facilitates precise liquid handling and individual analysis in distinct test areas. The device channels pretreated samples into the sedimentation chamber and the siphon valve, respectively. The former is employed for allergen extraction, while the latter is responsible for transporting purified allergens to the testing chamber. By utilizing existing optical detectors, parallel multiplex analysis of food allergens can be performed. Berti et al. discovered the utilization of active microfluidic valves in microfluidic devices. Different from passive valves, active valves exhibit the ability to withstand high-pressure loads, enabling precise fluid control and reusability [72]. Active valves operate reactions on demand and require manual operation to control the micropump drive and the magnetic drive. Lin et al. demonstrated a miniature fluid device based on electrochemical sensing and magnetic rod trapping for the rapid detection of food allergens [73] (Figure 5). The electrochemical sensor is connected to the corresponding program of the smartphone via Bluetooth and displays relevant information such as the detected allergen concentration and time [9]. Recently, Pan et al. developed a portable microfluidic paper-based analysis device (μ-PAD) combined with electrochemical techniques, which introduces black phosphorus (BP)–Au nanocomposites to amplify the signal by increasing the electron transfer rate at the electrode interface for an efficient and sensitive detection of Peanut allergen Ara h1 [74]. Magnetic rods serve as fully integrated microfluidic devices, eliminating the need for precision pipettes and facilitating allergen analysis in food. Furthermore, Huang et al. showcased an integrated microfluidic system equipped with an air-operated micropump for automated immunoassay IgE detection [75,76]. This system streamlines the process of allergen detection by automating multiple immunoassay steps, including incubation, hybridization, reagent transport, and washing. Magneto-chemical systems integrated with microfluidics can also detect food allergens in peanuts, rice dumplings, wheat, milk, and eggs. 

In addition to the aforementioned active strategy, the utilization of capillary power as a passive mode is a significant approach for the integration of paper-based chips. The initial demonstration of microfluidic paper analytical equipment was conducted by Martinez in 2007 [77]. The composition of this equipment involves cellulose, making it a desirable material due to its lightweight nature, biodegradability, and ease of manufacturing. Furthermore, paper exhibits strong plasticity is more suitable for food safety applications, and has a lower detection background compared to other substrates used in microfluidic equipment such as glass, silicon, and plastic. The abundance of hydroxyl groups and a small number of carboxyl groups on the surface of paper fibers make it susceptible to chemical denaturation and amenable to various processing techniques such as cutting, folding, and stacking. These characteristics also facilitate photometric detection [8]. Microfluidic devices can be designed in either 2D or 3D modes, without the need for external power sources. The capillary channels within these devices can be harnessed using their intrinsic capillary capacity, providing an effective method for pretreating food matrices and enabling allergen detection [78,79,80]. Capillary motility serves as a passive mode within the integrated strategy, harnessing the capabilities of capillary power. This mode exploits the vector transport and liquid diversion properties to automatically direct fluids to different areas. The pressure difference on the liquid surface, driven by capillary power, is generated by the hydrophilic action of surface glass capillaries or microfluidic microchannels. Paper-based microfluidics enable fluid control through the construction of paper-based valves, allowing for the cutting off and connection of different zones [81].

With the advancement of the social economy, there has been a significant improvement in people’s living standards, resulting in an increased demand for high-quality food ingredients. In a study conducted by Li et al., cellulose filter paper was utilized as a substrate to construct a micro-laboratory on paper. This innovative approach combined colorimetric analysis technology, leading to the establishment of a novel method for the detection of glucose content in fruits [82]. Similarly, Hossain et al. employed inkjet printing technology to fabricate a paper chip on filter paper, enabling the detection of organophosphorus pesticide residues in food and beverages through the examination of ace-phthalein cholinesterase activity [83]. Similarly, Deng et al. constructed a method combining ammonium molybdate spectrophotometry with a microfluidic paper-based platform to analyze and detect the organophosphorus pesticide trichlorfon [84]. Furthermore, Yang et al. detected the pesticide trichlorfon on a paper-based platform, and the limit of detection was 00406 mg/L [85]. In another study, Xue et al. employed monoclonal antibodies and rabbit anti-Salmonella enteritidis polyclonal antibodies as capture and detection antibodies, respectively [86]. By employing traditional bispecific antibody sandwich immunoassay technology and paper-based microfluidic chip technology, a paper-based microfluidic chip was successfully developed for the rapid detection of Salmonella [87]. Additionally, Zhang et al. utilized a fluorescently labeled single-stranded DNA (ssDNA) functionalized graphene oxide sensor to create a low-cost and simple paper-based microfluidic device [88]. This device demonstrated the capability to simultaneously determine multiple chemical contaminants in food. Recently, Zhang et al. developed a novel nano-fluorescent probe with a suitable ratio by combining the excellent properties of gold nanoparticles and carbon dots. The paper probe is used to detect glyphosate residues (pesticides) and identify them in real time using smartphones and the tone detector app. This method can complete the detection reaction within 2 s and is suitable for sensing in different situations as an enzyme-free probe. In addition, the method uses a color detection app and an integrated portable smartphone tool to quantitatively identify test results [89,90]. Furthermore, the utilization of colorimetry, fluorescence, and surface-enhanced Raman scattering analysis methods on the paper chip platform has exhibited rapid, convenient, and efficient performance. These advancements have demonstrated promising potential in the field of rapid detection of food safety [91].

**Table 1:** Summary of microfluidic paper chips in food safety testing.

Target	Chip Material	Detection Method	Limits of Detection (LOD)	Real Sample Application	Property	Reference
nitrite	paper	electrochemical analysis	1.06 μM	—	low cost, simple, and reproducible	[46]
nitrite	paper	colorimetric analysis	19.2 mg/kg	pork	high selectivity, sensitivity, environment-friendly, and suitable for on-site measurement	[60]
glucose	paper	electrochemical analysis	0.12 μM	—	high sensitivity and good selectivity	[61]
Ascorbic acid	paper	colorimetric analysis	0.406 μmol/L	—	reliable and sensitive	[64]
heavy metal ions	paper	fluorescence	0.035 µg/L, 0.056 µg/L	water	good linear relationship	[65]
2,4-dichloro phenoxy acetic acid	paper	fluorescence	90 nM	soybean sprouts	fast response, high precision, practical availability, and good reproducibility	[66]
*E. coli* and Enterococcus	paper	electrochemical and colorimetric analysis	81 μΜ and 119 μM	—	fast response, and high sensitivity	[67]
*E. coli*	paper	fluorescence and colorimetric analysis	100 CFU/mL and 44 CFU/mL	—	rapid, portable, and sensitive	[68]
methyl parathion	paper	SERE	0.011 μg/cm^2^	apple	low cost and on-site inspection	[69]
pesticide thiram	paper	SERE	0.024 ppm, 600 ng/cm^2^	water and apple juice, apple peel	high sensitivity and environment-friendly	[70]
peanut allergen Ara h1	paper	electrochemical analysis	11.8 ng/mL	cookies, milk, and bread	specificity, sensitivity, and good stability	[74]
sulfite	paper	colorimetric analysis	78 μM	wines	environment-friendly and high sensitivity	[79]
allergens	paper	colorimetric analysis	0.246 KUA/L	—	efficient, accurate, and sensitive	[80]
glucose	paper	colorimetric analysis	3.12 mM	fruits	low cost and highly sensitive	[82]
paraoxon	paper	colorimetric analysis	1 μM	milk and apple juice	environment-friendly, and high selectivity	[83]
trichlorfon	paper	spectrophotometric	1.65 mg/mL	vegetable	rapid and sensitive	[84]
trichlorfon	paper	colorimetric analysis	0.0406 mg/L	—	repeatability, specificity, good stability	[85]
Salmonella	paper	electrochemical analysis	5 cells/mL	apple juice	simple and accessible	[87]
Hg^2+^, Ag^+^ and NEO	paper	fluorescence	121 nM, 47 nM, 153 nM	—	low cost, simple, and multiplexed	[88]
glyphosate	paper	fluorescence	4.19 nM	—	rapid, on-site inspection	[89]
Pb	paper	fluorescence	18.3 nM	—	rapid, on-site inspection, and high selectivity	[90]

## 6. Conclusions and Future Perspectives

Food safety and pollution issues directly related to human health and social harmony have garnered global attention for an extended period. This review paper examines the research progress in rapid detection methods for food safety utilizing paper-based analysis platforms. It delves into the preparation strategy, surface functionalization modification, and analytical sensing methods of paper-based devices. Notably, the advancement of paper-based surface modification technology has facilitated the successful development of various analytical methods on these platforms, with analysis techniques based on optical, electrochemical, and Raman spectroscopy signals being extensively explored. The implementation of diverse analysis methods on paper-based platforms has not only enhanced the efficiency of food safety testing but also has transitioned from qualitative to semi-quantitative analysis, along with highly sensitive and selective detection. Since lateral flow immunoassay commercialization for home pregnancy testing, paper-based devices have gained recognition for their simplicity, affordability, and potent microfluidic capabilities. In recent years, food adulteration has become a major concern in the food field. A paper-based lateral flow assay strip has been developed to detect adulteration in foods such as milk. The paper-based sensor uses specific DNA probes to amplify species-specific DNA sequences and the visual properties of gold nanoaggregates [92]. Similarly, Muthukumar et al. developed a paper-based microfluidic lab-on-a-chip to detect palm oil contamination in sunflower oil. Combined with the colorimetric detection method, it can be visually observed whether there is adulteration in edible oil [93]. Despite their remarkable success in rapid inspection, it is crucial to address certain parameters that influence the analytical performance of paper-based devices to cater to the specific requirements of different inspection fields. 

Firstly, the combination of paper-based microfluidic devices with pre-enrichment methods and their applicability in field applications have not received adequate attention. The inclusion of pre-enrichment steps significantly enhances the sensitivity as compared to methods without pre-enrichment. Hence, the development of novel strategies heavily reliant on advanced material synthesis, new sensing mechanisms, multiple detection modes, reliable micromachining methods/standards, and simplified detection procedures is imperative. Secondly, the integration of nanomaterials and nanotechnology into paper-based devices presents an opportunity to improve their analytical performance, encompassing sensitivity, selectivity, reproducibility, stability, and multiplexing capabilities. For instance, employing carbon nanomaterials with high conductivity and specific surface area as electrode materials or modifiers considerably enhances the sensitivity of paper-based device detection. The compatibility with dual-mode detection modes, such as colorimetric-fluorescence and electrochemical-optical inspection, ensures reliable and accurate analysis with paper-based devices. Lastly, the challenge lies in avoiding the reliance on expensive instruments like benchtop potentiostats for reading electrochemical signals. One alternative is to connect smartphones to dedicated circuit boards as a data transmission source for analyzing paper-based devices. Furthermore, further endeavors should concentrate on the development of instrumentless technology and non-device-based readings, which can be assisted by artificial intelligence [94]. Despite meticulous searching, it is undeniable that there may have been inadvertent omissions of essential papers. Ultimately, we hope that our review’s insights inspire readers to conceive more methods and ideas for paper-based devices employed in the realm of food safety.

## Data Availability

The data presented in this study are available upon request from the corresponding author.
